# Comparative Efficacy of Therapeutics for Chronic Cancer Pain: A Bayesian Network Meta-Analysis

**DOI:** 10.1200/JCO.18.01567

**Published:** 2019-04-02

**Authors:** Rongzhong Huang, Lihong Jiang, Yu Cao, Hongli Liu, Minsheng Ping, Wei Li, Yu Xu, Jie Ning, Yuqing Chen, Xiaojing Wang

**Affiliations:** ^1^ The First People’s Hospital of Yunnan Province, Kunming, People’s Republic of China; ^2^ First Affiliated Hospital, Bengbu Medical College, Bengbu, People’s Republic of China; ^3^ Chuangxu Institute of Lifescience, Chongqing, People’s Republic of China

## Abstract

**PURPOSE:**

Opioids are the primary choice for managing chronic cancer pain. However, many nonopioid therapies are currently prescribed for chronic cancer pain with little published evidence comparing their efficacy.

**METHODS:**

Electronic databases were searched for randomized controlled trials (RCTs) comparing any systemic pharmaceutical intervention and/or combination thereof in treating chronic cancer pain. The primary outcome was global efficacy reported as an odds ratio (OR). The secondary outcome was change in pain intensity reported as a standardized mean difference (SMD).

**RESULTS:**

We included 81 RCTs consisting of 10,003 patients investigating 11 medication classes. Most RCTs (80%) displayed low risk of bias. The top-ranking classes for global efficacy were nonopioid analgesics (network OR, 0.30; 95% credibility interval [CrI], 0.13 to 0.67), nonsteroidal anti-inflammatory drugs (network OR, 0.44; 95% CrI, 0.22 to 0.90), and opioids (network OR, 0.49; 95% CrI, 0.27 to 0.86), whereas the top-ranked interventions were lidocaine (network OR, 0.04; 95% CrI, 0.01 to 0.18; surface under the cumulative ranking curve analysis [SUCRA] score, 98.1), codeine plus aspirin (network OR, 0.22; 95% CrI, 0.08 to 0.63; SUCRA score, 81.1), and pregabalin (network OR, 0.29; 95% CrI, 0.08 to 0.92; SUCRA score, 73.8). In terms of reducing pain intensity, we found that no class was superior to placebo, whereas the following top-ranked interventions were superior to placebo: ziconotide (network SMD, −24.98; 95% CrI, −32.62 to −17.35; SUCRA score, 99.8), dezocine (network SMD, −13.56; 95% CrI, −23.37 to −3.69; SUCRA score, 93.5), and diclofenac (network SMD, −11.22; 95% CrI, −15.91 to −5.80; SUCRA score, 92.9).

**CONCLUSION:**

There are significant differences in efficacy among current regimens for chronic cancer pain. Our evidence suggests that certain nonopioid analgesics and nonsteroidal anti-inflammatory drugs can serve as effectively as opioids in managing chronic cancer pain.

## INTRODUCTION

Chronic pain remains a common symptom among patients with cancer with metastatic solid tumors.^1^ Clinical surveys suggest that undertreatment or inappropriate treatment of chronic cancer pain by practicing oncologists remains common.^1^ Therefore, effective chronic pain management remains a pressing clinical challenge for oncologists.^1^ Currently, opioids are the primary choice for chronic cancer pain, and their use in this regard has been supported by several authorities.^2-4^ In terms of therapeutic approach, the WHO analgesic ladder recommends opioid therapy on the basis of pain intensity (ie, no drug for no pain [step 0] ratcheting up to strong opioids [step III] for severe chronic pain).^5^ Several previous trials have already compared the efficacy and safety of various opioid regimens in patients with chronic cancer pain and have found their analgesic efficacy and safety to be largely similar.^6-8^

Although this previous evidence suggests that opioids share similar characteristics and efficacy in patients with chronic cancer pain, there are many nonopioid therapies (eg, nonsteroidal anti-inflammatory drugs [NSAIDs], nonopioid analgesics, antidepressants) that are currently being prescribed to treat chronic cancer pain.^9,10^ Moreover, the use of these therapeutics as adjuvants in conjunction with opioid therapy has been shown to produce interactions that affect the opioid response.^7^ However, there is little published evidence comparing the efficacy of these various therapeutic regimens in patients with chronic cancer pain. To address this question, Bayesian network meta-analysis enables a comprehensive analysis through integrating all available direct and indirect evidence across multiple trials to compare various therapeutic regimens.^11^ Therefore, the aim of this Bayesian network meta-analysis will be to comprehensively evaluate the effectiveness of various therapeutic regimens for chronic cancer pain.

## METHODS

This meta-analysis adheres to the guidelines provided by the Preferred Reporting Items for Systematic Reviews and Meta-Analyses report (Appendix Table A1, online only). This meta-analysis is based on summary data.

### Literature Search

MEDLINE, Embase, and the Cochrane Central Register of Controlled Trials were searched for randomized controlled trials (RCTs) from 1970 to the present (updated August 2018) using the targeted search strategy detailed in the Data Supplement. No language restrictions were applied on searches. Reference lists were searched for additional records.

### Study Selection

Two coauthors (R.H. and Y.X.) independently scanned relevant records to determine their eligibility for inclusion. In the event of disagreement, rechecking the original article followed by discussion was used to achieve consensus. The following inclusion criteria were applied: RCTs of adult patients with cancer (age 18 years or older) comparing any systemic pharmaceutical intervention and/or combination thereof (including oral, transdermal, intravenous, and subcutaneous routes) for chronic cancer pain. All included patients within each RCT must have a positive cancer diagnosis. For RCTs investigating adjuvant therapeutics, RCTs with participants receiving background analgesics were allowed only if the dosing of background analgesia remained stable during the study period. Because patients with chronic cancer pain commonly suffer from episodes of acute pain termed breakthrough pain that are superimposed on their background chronic pain,^12^ RCTs assessing breakthrough pain were excluded to focus the analysis on chronic pain. Non-RCTs, case studies and case series, nonhuman studies, conference abstracts and summaries, and reviews or meta-analyses were excluded from consideration.

### Data Extraction

Two coauthors (Y.X. and Y.Cao) independently extracted relevant data parameters. In the event of disagreement, rechecking of the original article followed by discussion was used to reach a consensus. The following data parameters were extracted: name of primary author, country(s) of study, RCT design, patient population under study, number of participants in each arm, patient age (mean or median and standard deviation [SD] or range if available), patient sex, characteristics of pharmaceutical intervention (dosage and duration of therapy) in each arm, co-interventions across both arms, pain scale, follow-up duration, outcome type (pain or function), and outcome measures for each arm.

Pain data were extracted at the point of treatment termination or the final study time point, whichever was later. For crossover RCTs, data were solely extracted from the first period as a result of potential carryover effects. When needed, dispersion effects were approximated from the figures provided. Missing data, such as SD, were estimated from the published data.^13^ Where mean value was unavailable but the median was reported, median was used. If SD was missing but the baseline SD was reported, this was substituted for the missing SD. Intent-to-treat data were used when available.

### Risk-of-Bias Assessment

The risk of bias of included RCTs was assessed using a modified version of the Cochrane Collaboration’s Risk-of-Bias Tool.^14^ Two coauthors (X.W. and R.H.) independently performed the risk-of-bias assessment on all included RCTs. In the event of disagreement, rechecking of the original article followed by discussion was used to reach a consensus.

### Quality-of-Evidence Assessment

The quality or certainty of the evidence for each comparison was assessed using the Grading of Recommendations Assessment, Development, and Evaluation (GRADE) approach. For each comparison, the direct estimate (if available), the indirect estimate (if available), and the network estimate with the GRADE quality or certainty were assessed as previously described.^15-19^ The five items included in the GRADE assessment were study limitations, imprecision, inconsistency, indirectness, and publication bias. The confidence in each effect estimate was downgraded according to our assessments of these five items. The judgment of precision was based on the width of the credibility interval (CrI) around the effect estimate, the effect estimate, and the sample size, as previously described.^20^

### Outcomes

The prespecified primary outcome was global efficacy (or overall response). Global efficacy was analyzed as a binary outcome (ie, treatment success *v* failure) and reported as an odds ratio (OR) with the associated 95% CrI. The OR was calculated by taking the odds of treatment failure with one active treatment or medication class and dividing this value by the odds of treatment failure with placebo (or another active treatment or medication class). Therefore, treatment success was defined as an OR (including the associated 95% CrI) falling under unity (1.0). For trials reporting both the provider’s and patient’s global efficacy, the patients’ perceived effect was used.

The prespecified secondary outcome was change in pain intensity as measured by a standardized pain scale. Specifically, pain intensity had to be assessed using single scales that could be linearly transformed to a standardized 100-point scale; therefore, pain scale data were not interconverted nor was a reference pain scale instrument used. The effect size was calculated as follows. First, the difference between pain intensity with one active treatment or medication class and that with placebo (or another active treatment or medication class) was calculated, and then the standardized mean difference (SMD) in pain intensity with the active treatment or medication class minus that with placebo (or another active treatment or medication class) was calculated. Therefore, a negative SMD value denotes an improvement in pain relief.

### Statistical Analysis

The network meta-analyses were performed with a Bayesian hierarchical random effects model using WinBUGS (version 1.4.3; MRC Biostatistics Unit, Cambridge, United Kingdom).^21^ The detailed statistical methods are provided in the Data Supplement. To measure the consistency of the effect size (OR and SMD), pairwise meta-analyses were performed with a DerSimonian and Laird random effects model to calculate the pooled estimates of OR and SMD with 95% CIs of direct comparisons between placebo and a medication class or individual treatment using STATA (version 10.0; StataCorp, College Station, TX).^13^ Heterogeneity of treatment effects across studies was assessed using the I^2^ statistic (ie, the measure of interstudy variation as a result of heterogeneity rather than chance), the Cochrane Q test (ie, a measure of the weighted sum of squared differences between individual study effects and the pooled effect), and the τ^2^ statistic (ie, a measure of between-study variance in the random effects model).^13^ Effect modifier analyses were performed to detect potential sources of clinical and methodologic heterogeneity within each network meta-analysis. Inconsistency analyses were performed using both design-by-treatment and loop-specific approaches.^22^

## RESULTS

### Study Selection

We identified 9,055 nonduplicate records, of which 81 were RCTs consisting of 10,003 total participants (Data Supplement). The characteristics and results of the included studies are detailed in the Data Supplement. The included RCTs encompassed 11 unique medication classes; the abbreviations for the medication classes and their constituent individual treatments are listed in Table 1. The mean age of participants ranged from 25.0 to 71.5 years, with a median age of 58.4 years, and the proportion of male participants ranged from 17.1% to 86.0%, with a median percentage of 51.7%. The follow-up duration extended from 6 hours to 1 year, with a median duration of 14 days (interquartile range, 7 to 15 days).

**TABLE 1.**
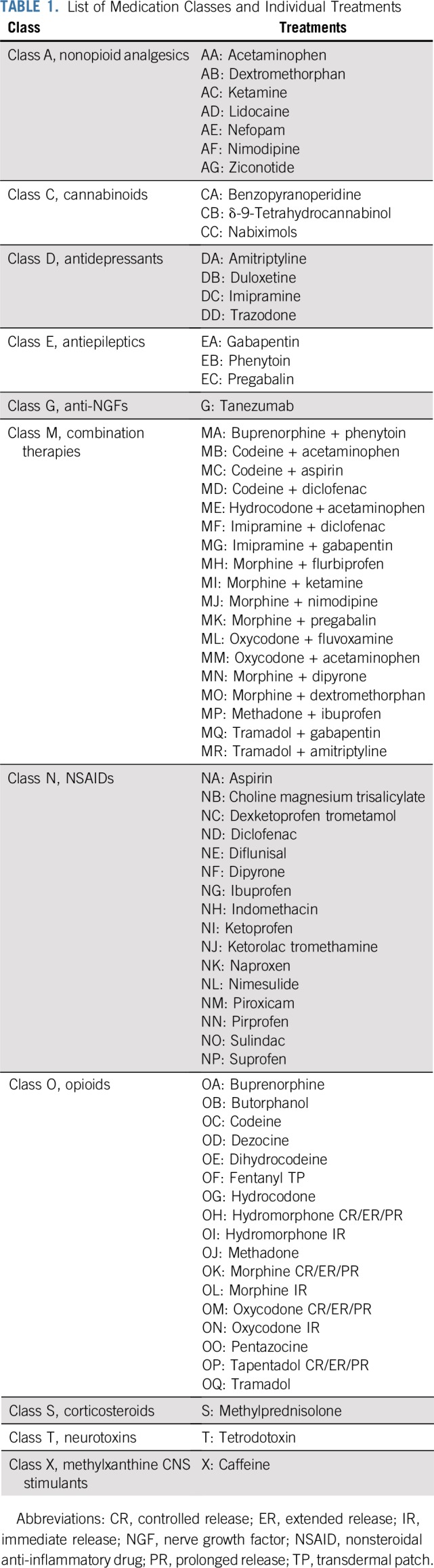
List of Medication Classes and Individual Treatments

### Risk-of-Bias and Quality-of-Evidence Assessment

We found that the vast majority of included RCTs had low risk of bias, with 80.2% possessing an overall rating of 7 of 12 or greater (Data Supplement). Analyzing by individual risk-of-bias categories, 10 of the 12 categories displayed a low risk of bias in greater than 60% of the included studies (Data Supplement). Only two categories, random assignment and allocation concealment, displayed a high risk of bias in greater than 50% of the included studies (Data Supplement).

Assessing the global efficacy analysis using the GRADE approach revealed suspected imprecision and publication bias for both individual treatments and medication classes, extending across almost all indirect or mixed comparisons (Data Supplement). Assessing the pain intensity analysis using the GRADE approach revealed suspected imprecision and incoherence for both individual treatments and medication classes (Data Supplement). Suspected publication bias was found among many comparisons, especially for medication classes.

### Global Efficacy Meta-Analysis by Medication Class

**FIG 1.**
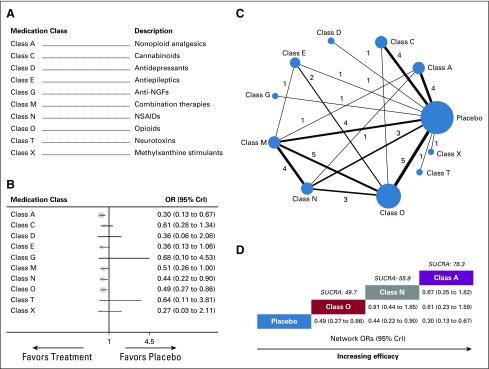
Meta-analysis of global efficacy by medication class. (A) Descriptions of medication classes included in this meta-analysis. (B) Forest plot of the network meta-analysis comparing the global efficacy of each medication class against placebo. (C) Network plot showing comparisons in global efficacy between nodes (blue circles), each representing a unique medication class or placebo. The size of each node is proportional to total number of randomly assigned participants receiving the medication class. The width of each connecting line is proportional to the number of trial-level comparisons between the two nodes, which is noted next to each line. (D) Schematic detailing the most globally efficacious medication classes according to surface under the cumulative ranking curve analysis (SUCRA). CrI, credibility interval; NGF, nerve growth factor; NSAID, nonsteroidal anti-inflammatory drug; OR, odds ratio.

A total of 31 RCTs were included in our global efficacy meta-analysis by medication class. There were 29 two-arm studies and two three-arm studies (Data Supplement). A total of 10 medication classes were included (Fig 1A). Placebo (23 RCTs), opioids (class O; 13 RCTs), and combination therapies (class M; nine RCTs) were the three most commonly investigated classes (Data Supplement). The pairwise meta-analysis comparing each medication class against placebo revealed that nonopioid analgesics (class A; OR, 0.23; 95% CI, 0.08 to 0.56) were significantly superior to placebo (Data Supplement). All other classes were statistically equivalent to placebo (Data Supplement).

A total of 11 nodes were included in our global efficacy network meta-analysis, with each node representing a unique medication class or placebo (Fig 1C). The nodes with the most direct interactions in the network were placebo (25 interactions), opioids (class O; 14 interactions), and combination therapies (class M; 13 interactions; Fig 1C). The model fit was good (Data Supplement). Pooled network OR values indicate that nonopioid analgesics (class A; network OR, 0.30; 95% CrI, 0.13 to 0.67), NSAIDs (class N; network OR, 0.44; 95% CrI, 0.22 to 0.90), and opioids (class O; network OR, 0.49; 95% CrI, 0.27 to 0.86) showed significantly superior global efficacy compared with placebo (Data Supplement). Surface under the cumulative ranking curve analysis (SUCRA) analysis provided a ranking of each medication class according to its global efficacy (Data Supplement). The resulting top-ranked classes for global efficacy were nonopioid analgesics (class A; SUCRA score, 76.3), NSAIDs (class N; SUCRA score, 55.9), and opioids (class O; SUCRA score, 49.7; Fig 1D).

τ^2^ estimates revealed significant statistical heterogeneity (> 50%) for the class A and class M analyses, but no significant loop inconsistency was observed (Data Supplement). There was no evidence of publication bias (Data Supplement).

### Global Efficacy Meta-Analysis by Individual Treatment

**FIG 2.**
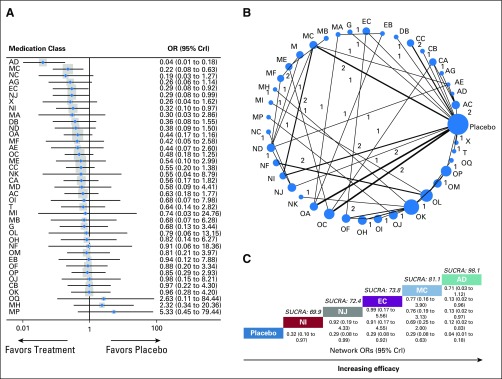
Meta-analysis of global efficacy by individual treatment. (A) Forest plot of the network meta-analysis comparing the global efficacy of each treatment against placebo. (B) Network plot showing comparisons in global efficacy between nodes (blue circles), each representing a unique intervention or placebo. The size of each node is proportional to total number of randomly assigned participants receiving the treatment. The width of each connecting line is proportional to the number of trial-level comparisons between the two nodes, which is noted next to each line. (C) Schematic detailing the most globally efficacious treatments according to surface under the cumulative ranking curve analysis (SUCRA). AC, ketamine; AD, lidocaine; AE, nefopam; AG, ziconotide; CA, benzopyranoperidine; CB, δ-9-tetrahydrocannabinol; CC, nabiximols; CrI, credibility interval; DB, duloxetine; EB, phenytoin; EC, pregabalin; G, tanezumab; MA, buprenorphine plus phenytoin; MB, codeine plus acetaminophen; MC, codeine plus aspirin; MD, codeine plus diclofenac; ME, hydrocodone plus acetaminophen; MF, imipramine plus diclofenac; MH, morphine plus flurbiprofen; MI, morphine plus ketamine; MP, methadone plus ibuprofen; NC, dexketoprofen trometamol; ND, diclofenac; NF, dipyrone; NG, ibuprofen; NI, ketoprofen; NK, naproxen; OA, buprenorphine; OC, codeine; OF, fentanyl transdermal patch; OH, hydromorphone controlled release/extended release/prolonged release; OI, hydromorphone immediate release; OJ, methadone; OK, morphine controlled release/extended release/prolonged release; OL, morphine immediate release; OM, oxycodone controlled release/extended release/prolonged release; OP, tapentadol controlled release/extended release/prolonged release; OQ, tramadol; OR, odds ratio; T, tetrodotoxin; X, caffeine.

A total of 47 RCTs were included in our global efficacy meta-analysis by individual treatment. There were 42 two-arm studies and five three-arm studies (Data Supplement). A total of 39 unique treatments were included (Fig 2A). Placebo (21 RCTs), morphine extended release (ER; eight RCTs), and morphine immediate release (IR; five RCTs) were the three most commonly investigated interventions (Data Supplement). The pairwise meta-analysis comparing each intervention against placebo revealed that lidocaine (OR, 0.04; 95% CI, 0.01 to 0.20) and codeine plus aspirin (OR, 0.19; 95% CI, 0.05 to 0.77) were significantly superior to placebo (Data Supplement). All other treatments were statistically equivalent to placebo (Data Supplement).

A total of 40 nodes were included in our global efficacy network meta-analysis, with each node representing a unique intervention or placebo (Fig 2B). The nodes with the most direct interactions in the network were placebo (23 interactions), morphine ER (eight interactions), and codeine plus aspirin (five interactions; Fig 2B). The model fit was good (Data Supplement). Pooled network OR values indicate that lidocaine (network OR, 0.04; 95% CrI, 0.01 to 0.18), codeine plus aspirin (network OR, 0.22; 95% CrI, 0.08 to 0.63), pregabalin (network OR, 0.29; 95% CrI, 0.08 to 0.92), ketorolac tromethamine (network OR, 0.29; 95% CrI, 0.08 to 0.99), and ketoprofen (network OR, 0.32; 95% CrI, 0.10 to 0.97) showed significantly superior global efficacy over placebo (Data Supplement). SUCRA analysis provided a ranking of each medication class according to its global efficacy (Data Supplement). The top-ranked interventions for global efficacy were lidocaine (SUCRA score, 98.1), codeine plus aspirin (SUCRA score, 81.1), and pregabalin (SUCRA score, 73.8; Fig 2C).

τ^2^ estimates indicate no significant statistical heterogeneity, and no significant loop inconsistency was observed (Data Supplement). There was evidence of significant publication bias (Data Supplement).

### Pain Intensity Meta-Analysis by Medication Class

**FIG 3.**
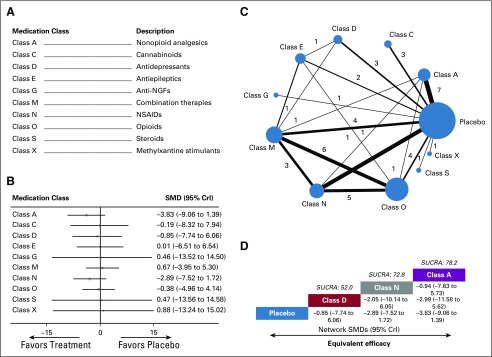
Meta-analysis of pain intensity by medication class. (A) Descriptions of medication classes included in this meta-analysis. (B) Forest plot of the network meta-analysis comparing changes in pain intensity for each medication class against placebo. (C) Network plot showing comparisons in pain intensity changes between nodes (blue circles), each representing a unique medication class or placebo. The size of each node is proportional to total number of randomly assigned participants receiving the medication class. The width of each connecting line is proportional to the number of trial-level comparisons between the two nodes, which is noted next to each line. (D) Schematic detailing the most efficacious medication classes in terms of reducing pain intensity according to surface under the cumulative ranking curve analysis (SUCRA). CrI, credibility interval; NGF, nerve growth factor; NSAID, nonsteroidal anti-inflammatory drug; SMD, standardized mean difference.

A total of 45 RCTs were included in the meta-analysis of pain intensity by medication class. There were 42 two-arm studies, two three-arm studies, and one four-arm study (Data Supplement). A total of 10 classes were included (Fig 3A). Placebo (31 RCTs), opioids (class O; 15 RCTs), and combination therapies (class M; 13 RCTs) were the three most commonly investigated classes (Data Supplement). The pairwise meta-analysis comparing each medication class against placebo revealed that no medication class was significantly superior to placebo (Data Supplement).

A total of 11 nodes were included in our pain intensity network meta-analysis, with each node representing a unique medication class or placebo (Fig 3C). The nodes with the most direct interactions in the network were placebo (32 interactions), opioids (class O; 17 interactions), and combination therapies (class M; 17 interactions; Fig 3C). The model fit was good (Data Supplement). Pooled network SMD values indicate that no medication class significantly improved pain intensity when compared with placebo (Data Supplement). SUCRA analysis provided a ranking of each medication class according to its efficacy in reducing pain intensity (Data Supplement). Although all classes were equivalent to placebo, the top-ranked classes for reducing pain intensity were nonopioid analgesics (class A; SUCRA score, 78.2), NSAIDs (class N; SUCRA score, 72.8), and antidepressants (class D; SUCRA score, 52.0; Fig 3D).

τ^2^ estimates indicate significant statistical heterogeneity (> 50%) for several classes (ie, A, C, D, M, N, and O), but no significant loop inconsistency was observed (Data Supplement). There was no evidence of publication bias (Data Supplement).

### Pain Intensity Meta-Analysis by Individual Intervention

**FIG 4.**
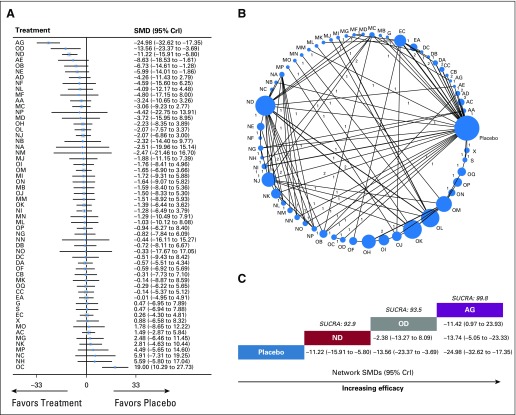
Meta-analysis of pain intensity by individual treatment. (A) Forest plot of the network meta-analysis comparing changes in pain intensity for each treatment against placebo. (B) Network plot showing comparisons in pain intensity changes between nodes (blue circles), each representing a unique intervention or placebo. The size of each node is proportional to total number of randomly assigned participants receiving the treatment. The width of each connecting line is proportional to the number of trial-level comparisons between the two nodes, which is noted next to each line. (C) Schematic detailing the most efficacious treatments in terms of reducing pain intensity according to surface under the cumulative ranking curve analysis (SUCRA). AA, acetaminophen; AC, ketamine; AD, lidocaine; AE, nefopam; AG, ziconotide; CB, δ-9-tetrahydrocannabinol; CC, nabiximols; CrI, credibility interval; DA, amitriptyline; DB, duloxetine; DC, imipramine; EA, gabapentin; EC, pregabalin; G, tanezumab; MB, codeine plus acetaminophen; MC, codeine plus aspirin; MD, codeine plus diclofenac; MF, imipramine plus diclofenac; MG, imipramine plus gabapentin; MI, morphine plus ketamine; MJ, morphine plus nimodipine; MK, morphine plus pregabalin; ML, oxycodone plus fluvoxamine; MM, oxycodone plus acetaminophen; MN, morphine plus dipyrone; MO, morphine plus dextromethorphan; MP, methadone plus ibuprofen; NA, aspirin; NB, choline magnesium trisalicylate; NC, dexketoprofen trometamol; ND, diclofenac; NE, diflunisal; NF, dipyrone; NG, ibuprofen; NH, indomethacin; NI, ketoprofen; NJ, ketorolac tromethamine; NK, naproxen; NL, nimesulide; NM, piroxicam; NN, pirprofen; NO, sulindac; NP, suprofen; OB, butorphanol; OC, codeine; OD, dezocine; OF, fentanyl transdermal patch; OH, hydromorphone controlled release/extended release/prolonged release; OI, hydromorphone immediate release; OJ, methadone; OK, morphine controlled release/extended release/prolonged release; OL, morphine immediate release; OM, oxycodone controlled release/extended release/prolonged release; ON, oxycodone immediate release; OP, tapentadol controlled release/extended release/prolonged release; OQ, tramadol; S, methylprednisolone; SMD, standardized mean difference; X, caffeine.

A total of 72 RCTs were included in the meta-analysis of pain intensity by individual treatment. There were 62 two-arm studies, six three-arm studies, three four-arm studies, and one nine-arm study (Data Supplement). A total of 57 unique treatments were included in this analysis (Fig 4A). Placebo (29 RCTs), morphine ER (12 RCTs), and morphine IR (12 RCTs) were the three most commonly investigated interventions (Data Supplement). The pairwise meta-analysis comparing each intervention against placebo revealed that ziconotide (SMD, −25.00; 95% CI, −29.00 to −21.00), dezocine (SMD, −2.00; 95% CI, −22.00 to −2.80), diclofenac (SMD, −25.00; 95% CI, −28.00 to −21.00), and lidocaine (SMD, −4.10; 95% CI, −7.50 to −0.72) were significantly superior to placebo (Data Supplement). All other treatments were statistically equivalent to placebo (Data Supplement).

A total of 58 nodes were included in our pain intensity network meta-analysis, with each node representing a unique intervention or placebo (Fig 4B). The interventions or nodes with the most direct interactions in the network were placebo (34 interactions), diclofenac (17 interactions), and morphine IR (14 interactions; Fig 4B). The model fit was good (Data Supplement). Pooled network SMD values indicate that ziconotide (network SMD, −24.98; 95% CrI, −32.62 to −17.35), dezocine (network SMD, −13.56; 95% CrI, −23.37 to −3.69), and diclofenac (network SMD, −11.22; 95% CrI, −15.91 to −5.80) improved pain intensity when compared with placebo (Data Supplement). SUCRA analysis provided a ranking of each intervention according to its efficacy in reducing pain intensity (Data Supplement). The top-ranked interventions for reducing pain intensity were ziconotide (SUCRA score, 99.8), dezocine (SUCRA score, 93.5), and diclofenac (SUCRA score, 92.9; Fig 4C).

τ^2^ estimates indicate no significant statistical heterogeneity; however, significant loop inconsistency was observed (Data Supplement). There was no evidence of publication bias (Data Supplement).

### Effect Modifier Analysis

The effect modifier analysis for the global efficacy outcome showed significant differences in terms of both age (*P* = .030) and follow-up duration (*P* = .008) among medication classes (Data Supplement). These factors were responsible for 37.37% and 40.15% of heterogeneity in the results, respectively. The effect modifier analysis for the pain intensity outcome revealed no significant differences among all modifiers tested (Data Supplement).

## DISCUSSION

Chronic cancer pain is one of the most prevalent symptoms affecting patients with cancer, with greater than one third of patients with cancer rating their pain as moderate to severe in nature.^23^ Unfortunately, the prevalence of chronic cancer pain has not significantly changed over the past decade relative to the preceding four decades, a phenomenon that has been attributed to undertreatment.^24^ Indeed, greater than one quarter of patients with cancer receive substandard care for their pain.^25,26^ In this network meta-analysis of 81 RCTs consisting of 10,003 patients with cancer, we compared the effectiveness of various therapeutic classes and individual treatments on chronic cancer pain. In terms of global efficacy, we found that nonopioid analgesics, NSAIDs, and opioids were the most effective classes, whereas the nonopioid analgesic lidocaine, the opioid-NSAID combination therapy of codeine plus aspirin, and the antiepileptic pregabalin were the most effective individual treatments. In terms of pain intensity, we found that no medication class significantly improved pain intensity compared with placebo, whereas the nonopioid analgesic ziconotide, the opioid dezocine, and the NSAID diclofenac were the most effective individual treatments. To our knowledge, this is the first network meta-analysis to comparatively evaluate the effectiveness of various therapeutic regimens for chronic cancer pain. Our findings indicate that there are significant differences in efficacy among current therapeutic regimens for chronic cancer pain. More importantly, in contrast to commonly held beliefs regarding the superiority of opioids as the mainstay of cancer pain therapy,^27^ our evidence suggests that certain nonopioid analgesics and NSAIDs can serve as effectively as opioid therapy in managing chronic cancer pain.

There have been previously published traditional meta-analysis that have focused on comparing a limited set of classes or individual therapies. The meta-analysis by Eisenberg et al^28^ of 25 cancer pain studies found that NSAIDs were as effective as weak opioids alone or in combination with nonopioid analgesics. However, for the most part, the vast majority of meta-analyses in this field have focused exclusively on comparing common opioid formulations (eg, oxycodone *v* morphine, transdermal fentanyl *v* morphine).^29,30^ In contrast to these meta-analyses, this network meta-analysis integrates a much broader base of published RCT evidence on chronic cancer pain to comprehensively evaluate several classes and individual therapies under one overarching analysis. This was accomplished by integrating direct and indirect comparisons in our model to enable formal comparisons between various classes and individual therapies. This work is of particular relevance to clinical practitioners, because our analysis reports explicit, quantitative comparisons between various drug classes and individual interventions for chronic cancer pain.

Appropriate drug selection is a major challenge in patients with advanced cancer, particularly in elderly individuals with polypharmacy.^31^ In terms of global efficacy, here we found that lidocaine, codeine plus aspirin, and the antiepileptic pregabalin were the most effective individual treatments for chronic cancer pain. On the basis of the reported evidence, we assessed the nonopioid analgesic lidocaine as a systemic intravenous therapy. Unfortunately, lidocaine possesses potential cardiotoxic effects, limiting its use to circumstances involving close observation and vital sign monitoring.^32^ Given our promising findings regarding intravenous lidocaine, future studies should focus on the transdermal and subcutaneous formulations of lidocaine that have been recently introduced for cancer patients.^33,34^ The highest ranking oral formulation, codeine plus aspirin, is an opioid-NSAID combination analgesic commonly used to control postoperative and postpartum pain.^35^ Notably, although codeine is approximately 10 times weaker than hydrocodone and approximately 15 to 20 times weaker than oxycodone according to the equianalgesia chart,^36^ we found that the combination of codeine plus aspirin was superior to stronger opioid formulations in terms of global efficacy. This suggests that strong opioids may not be necessary for adequate pain management in patients with chronic cancer pain. The next most effective formulation after codeine plus aspirin, pregabalin, is an antiepileptic medication that is also used to manage postoperative pain, neuropathic pain, and fibromyalgia.^37-39^ Notably, there is no opioid constituent in the pregabalin formulation, further supporting our conclusion that opioid therapy is not necessary for adequate pain management in patients with chronic cancer pain.

There are several limitations to this study. First, network meta-analyses, like all pooled analyses, should only combine the results of similar studies.^40^ Factors that drive nonstatistical heterogeneity (eg, differences in medications within drug classes, differences in study settings) are difficult to quantify.^40^ Therefore, we needed to make subjective assessments of which RCTs to pool. In the analyses by drug class, we made the assumption that drugs within each medication class were similar enough to justify pooling, which does not account for possible intra–drug class heterogeneity. Indeed, we found significant heterogeneity (τ^2^ > 50%) with several drug classes (global efficacy: classes A and M; pain intensity: classes A, C, D, M, N, and O), whereas we found no significant heterogeneity in the individual treatment-based network meta-analyses. Although our effect modifier analysis revealed that age and follow-up duration contributed to the observed heterogeneity in the global efficacy analysis, it is possible that intra–drug class heterogeneity may have contributed to the heterogeneity observed in the pain intensity analysis. Therefore, our findings with regard to the aforementioned drug classes should be cautiously interpreted in conjunction with our individual treatment-based findings.

Second, several individual drug comparisons included in our network meta-analyses (either to placebo or another drug) were only represented by one study, as can be observed in the network diagrams in Figures 2 and 4. As a result of the lower level of evidence, these results should be interpreted with more caution. Third, although our Cochrane risk-of-bias assessment showed that 80.2% of included RCTs displayed low risk of bias, the GRADE-based assessments revealed suspected imprecision in both network meta-analyses. In a clinical context, imprecision implies that CrIs are more likely to span effect regions that mandate treatment but also effect regions where treatment is not mandated.^20^ Therefore, imprecision is consequential for pain management interventions with serious adverse effects and/or costs^20^ (eg, opioids, antidepressants, antiepileptics) but is a less important factor for decisions regarding nonopioid analgesics and NSAIDs. Fourth, this network meta-analysis included many small-scale studies and is thus at risk for overestimating effect sizes, because journals are more likely to publish studies with large effect sizes.^41^ Indeed, we discovered evidence of significant publication bias in the global efficacy analysis by individual treatment. Therefore, the efficacies of lidocaine, codeine plus aspirin, pregabalin, ketorolac tromethamine, and ketoprofen relative to placebo may have been overestimated as a result of publication bias.

In conclusion, this network meta-analysis of 81 RCTs consisting of 10,003 patients with chronic cancer pain found that, in terms of global efficacy, nonopioid analgesics, NSAIDs, and opioids were the most effective medication classes, whereas the nonopioid analgesic lidocaine, the opioid-NSAID combination therapy codeine plus aspirin, and the antiepileptic pregabalin were the most effective individual treatments. In terms of pain intensity, we also found that the nonopioid analgesic ziconotide, the opioid dezocine, and the NSAID diclofenac were the most effective individual treatments. Our findings indicate that there are significant differences in efficacy among current therapeutic regimens for chronic cancer pain. Our evidence also suggests that certain nonopioid analgesics and NSAIDs can serve as effectively as opioid therapy in managing chronic cancer pain.

## Figures and Tables

**TABLE A1.**
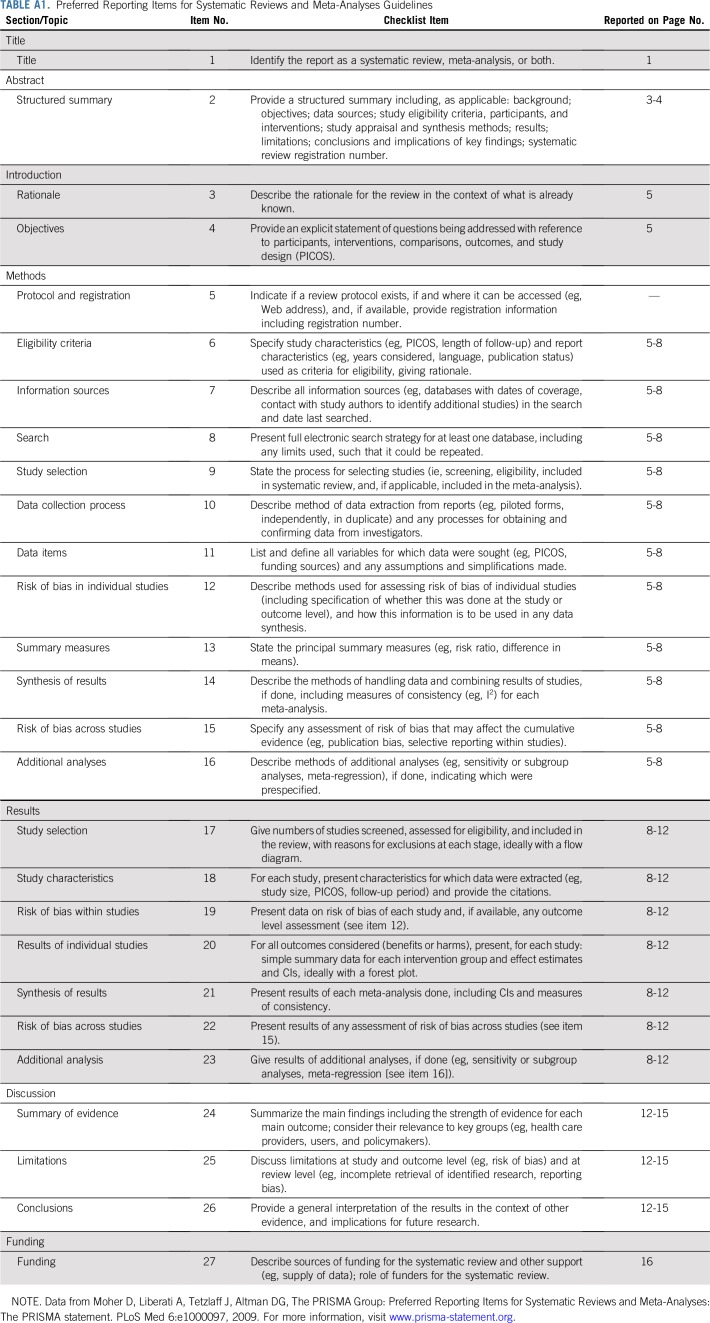
Preferred Reporting Items for Systematic Reviews and Meta-Analyses Guidelines
